# Democratising or disrupting diagnosis? Ethical issues raised by the use of AI tools for rare disease diagnosis

**DOI:** 10.1016/j.ssmqr.2023.100240

**Published:** 2023-06

**Authors:** Nina Hallowell, Shirlene Badger, Francis McKay, Angeliki Kerasidou, Christoffer Nellåker

**Affiliations:** a The Ethox Centre and Wellcome Centre for Ethics & Humanities, Nuffield Department of Population Health and Big Data Institute, University of Oxford, UK; b Illumina, Inc, Cambridge, UK; c Nuffield Department of Women's and Reproductive Health and Big Data Institute, University of Oxford, UK

**Keywords:** Computational phenotyping, Rare disease, Diagnosis, AI, Qualitative interviews

## Abstract

Computational phenotyping (CP) technology uses facial recognition algorithms to classify and potentially diagnose rare genetic disorders on the basis of digitised facial images. This AI technology has a number of research as well as clinical applications, such as supporting diagnostic decision-making. Using the example of CP, we examine stakeholders’ views of the benefits and costs of using AI as a diagnostic tool within the clinic. Through a series of in-depth interviews (n ​= ​20) with: clinicians, clinical researchers, data scientists, industry and support group representatives, we report stakeholder views regarding the adoption of this technology in a clinical setting. While most interviewees were supportive of employing CP as a diagnostic tool in some capacity we observed ambivalence around the potential for artificial intelligence to overcome diagnostic uncertainty in a clinical context. Thus, while there was widespread agreement amongst interviewees concerning the public benefits of AI assisted diagnosis, namely, its potential to increase diagnostic yield and enable faster more objective and accurate diagnoses by up skilling non specialists and thereby enabling access to diagnosis that is potentially lacking, interviewees also raised concerns about ensuring algorithmic reliability, expunging algorithmic bias and that the use of AI could result in deskilling the specialist clinical workforce. We conclude that, prior to widespread clinical implementation, on-going reflection is needed regarding the trade-offs required to determine acceptable levels of bias and conclude that diagnostic AI tools should only be employed as an assistive technology within the dysmorphology clinic.

## 1 Introduction

It is widely predicted that by, taking over certain mundane and/or time consuming tasks, the adoption of AI technology will revolutionise the practice of medicine (Topol, 2019). The use of AI diagnostic and screening tools, particularly those involved in image recognition, are increasingly seen as having the potential to produce efficiency gains and better healthcare (e.g. Davenport & Kalakota, 2019; Topol, 2019; National AI Strategy, 2022; HHS Artificial Intelligence Strategy, 2022). Other reports have itemized the ethical challenges this innovation presents, for example, ensuring the trustworthiness, transparency and accountability of AI-generated decisions (e.g. AI HLEG, 2019; Morley, Machado, et al., 2020; World Health Organization, 2021). While expert reports outline the economic, social and individual costs and benefits of deploying AI in healthcare much less is known about what those who use and develop this technology think about the introduction of specific AI tools in specific clinical contexts.

### 1.1 Empirical research on stakeholder views

A recent international survey of pathologists suggests this group is supportive of the introduction of a range of AI tools within the clinical workflow; the majority (72%) agreed that AI will increase diagnostic efficiency in this specialty (Sarwar et al., 2019). However, despite the general optimism reflected in their survey responses, a minority of respondents thought that the deployment of AI tools could result in the erosion of pathologists’ diagnostic skills and the majority (74%) thought AI should only be used as a decision-support tool, with humans, not AI, responsible for final diagnosis (Sarwar et al., 2019). These responses could be interpreted as indicating an acknowledgement that, unless their use is confined to an assistive role, AI tools may come to challenge human expertise. Ambivalence regarding the introduction of AI in clinical settings has also been noted in a couple of recent qualitative studies.

Lai et al., (2020) explored French stakeholders' understandings of AI and its implications for healthcare in semi-structured interviews and found that, from the clinicians’ point of view, a lack of explainability and understanding of AI was perceived as potentially damaging to the doctor-patient relationship. However, they also note that clinicians regarded AI technology as having the potential to improve healthcare, but only if staff are trained to use it properly. The AI researchers interviewed in the same study were similarly supportive of the adoption of clinical AI tools, but stressed they should only be used in an assistive role by suitably trained clinicians. Interviewees across the board stressed that collaboration between users, developers and regulators is important for safe clinical implementation (Lai et al., 2020).

One of the problems with Lai et al.‘s study is that AI was discussed in a generic sense and included people who were less familiar with this technology, although the study included a small group of radiologists who were more knowledgeable about these tools. A more recent study addresses the issue of familiarity by specifically focussing on the views of pathologists from two centres in the Netherlands, where the pathology workstream has been digitised and AI–based tools for image-based diagnosis are under development (Drogt et al., 2022). This study found that interviewees were similarly ambivalent about the implementation of AI tools in pathology. On the one hand, interviewees observed that AI can broaden pathology expertise and, like Lai and Sarwar et al.*s'* participants, suggested that the introduction of AI into the workflow could result in efficiency gains by: alleviating pathologists of more mundane tasks, triaging or pre-screening cases, supporting the diagnosis of complex cases and developing a standardized evidence base, which could be used in training junior pathologists. On the other hand, Drogt et al.*'s* interviewees observed that the costs and feasibility of implementation need to be properly assessed as do the roles and responsibilities of humans and AI in the pathology workflow, arguing that, independently of the type of role played by AI in diagnosis, human users must ultimately be seen as responsible for the final diagnostic decision. Drogt et al. offer a number of recommendations for the responsible implementation of AI which include: encouraging a pragmatic attitude to AI development, ensuring pathology staff are trained and supported in the use of AI and reflecting upon the impact of implementing AI systems on the roles and responsibilities of AI users.

While Drogt et al.*'s* (2022) study aimed to overcome the shortcomings of earlier work by focussing upon stakeholders working in centres involved in AI development, they note that their interviewees referred to AI in a number of ways:

“… as an umbrella term for image recognition tasks, other automated tasks, applications based on machine learning or deep learning, but also “simpler” algorithms and calculations. The picture that emerges from the interviews is of AI as a rather amorphous entity, which reflects the ambiguity of the term in broader scholarly and popular discourse. “(p. 1541ff)

They speculate that this may be due to the fact that some interviewees were less familiar with AI as, although their work involved digital pathology, they were not directly involved in AI development. The current study addresses the problem of familiarity by focussing upon a specific form of AI-based diagnostic technology and clinical/other stakeholders who are familiar with these tools.

Using the example of computational phenotyping technology and rare disease diagnosis, this paper presents data collected during an empirical bioethics study that sought to determine what stakeholders (clinicians and CP developers, industry and support group spokespersons) who are familiar with these specific tools regard as the ethical issues arising from their implementation in the clinic.

### 1.2 Computational phenotyping: refining the identification of rare disease phenotypes

Computational Phenotyping (CP) promises to refine and redefine the categorisation of rare genetic disorders (Ferry et al., 2014; Hsieh et al., 2022). This technology uses facial recognition (FR) algorithms to classify phenotypic features associated with different (rare) disorders. FR algorithms are trained using datasets of digitised facial images of individuals who have received a clinical or molecular diagnosis of a rare (often genetic) disease associated with particular dysmorphic facial features.

CP technology facilitates rare disease research in different ways. First, it enables researchers to identify new syndromes *via* precise and more comprehensive characterisation of facial phenotypes (Robinson, 2012). Second, it supports automated “matchmaking” for ultra-rare and unknown disorders (Zemojtel et al., 2014), thus, facilitating phenotypic comparisons between patients. Finally, it assists researchers in the classification of causative variants identified during genomic sequencing (Philippakis et al., 2015). In other words, FR technology can be used to identify individuals as having similar phenotypic features, thus suggesting a genotypic similarity, which, if confirmed, allows researchers to (re)classify genomic variants identified during sequencing (including variants of uncertain significance VUS) as disease-causing.

While CP is a valuable research tool, this technology also has clinical uses, specifically to aid the identification and diagnosis of rare genetic disorders. Clinical applications of CP have been developed in the commercial sector (eg. FACE2GENE an application developed by FDNA https://fdna.com/face2gene-for-geneticist-healthcare-providers/) and these are currently used in some genetics, paediatrics and specialist dysmorphology clinics.

### 1.3 Diagnosing rare disease: clinical expertise and molecular diagnostics

Dysmorphology is a medical subspeciality concerned with complex syndromes that impair physical development and are often associated with neurological impairments and cognitive delay. Diagnosis in dysmorphology involves identifying genotypic and phenotypic features, often facial features, as markers of an underlying syndrome, which may be caused by a single gene defect (e.g. NF1, Marfan Syndrome) or a chromosomal anomaly (e.g. Down Syndrome), or alternatively, as the result of a prenatal environmental insult (e.g. foetal alcohol spectrum disorder, ionising radiation exposure) (Reardon & Donnai, 2007).

Prior to the introduction of genotyping in the second half of the 20th century, the diagnosis of rare dysmorphological syndromes was based on clinical phenotyping using physical examinations, photographs and other types of clinical data (Shaw, 2003). Bearing in mind that many of these syndromes occur infrequently in the population, many clinicians’ experience of a particular syndrome is limited or based upon images in journals/books/online, rather than a physical examination. Consequently, clinical phenotyping of rare dysmorphological syndromes is regarded as an expert skill, which relatively few clinicians develop.

In her ethnographic study of the dysmorphology clinic Latimer (2013) details how the recent introduction of genetic testing impacted diagnostic work. She notes, that while for some families the introduction of molecular diagnostics foreshortened their diagnostic odyssey, there are still many families in which DNA testing fails to identify a known genetic disorder. Failure to obtain a molecular diagnosis in these cases does not mean, that the disorder does not have a genetic origin, but rather that researchers have not yet identified any genetic markers associated with the phenotype.

In cases where DNA testing is inconclusive, diagnosis depends on clinical phenotyping, which often takes place in case conferences where experts (e.g. clinical geneticists, paediatricians, paedatric neurologists) discuss the meaning of an individual's phenotypic markers and agree or not upon a diagnosis (see Shaw, 2003 and Latimer et al., 2006 for ethnographic analyses of diagnostic work in these case conferences). It has been suggested that the introduction of CP tools into the diagnostic pathway may supplement clinicians' phenotyping skills, which may circumvent the need for specialist case conferences and/or molecular testing in some instances and more generally expedite diagnosis for individuals and/or improve diagnostic yield by reducing diagnostic uncertainty (Nellåker et al., 2019).

### 1.4 The art of diagnosis and the issue of diagnostic uncertainty

“Diagnosis is medicine’s primary classification tool. It determines treatment and prognosis, allocates resources, differentiates lay from professional, and provides a hierarchy of authority within the professions” (Jutel, 2021: 2).

As Jutel (2021) notes, diagnosis plays a fundamental role in medical practice; the identification and classification of bodily symptoms as markers of disease is important for many medical tasks, including: providing aetiological explanations, treatment decision-making and prognosticating. The act of diagnosis typically involves the resolution of uncertainty, deciding whether observed symptoms are evidence for a particular underlying disease or not (Jutel, 2021; Topol, 2019). In many cases, but not all, this uncertainty is resolved and patients are provided with a diagnostic label and associated treatment plan. However, there are cases where a diagnosis is not forthcoming and diagnostic uncertainty persists. In addition to rare genetic disorders, which are the subject of this paper, examples of continuing diagnostic uncertainty include: chronic pain, chronic fatigue, endometriosis and more recently, long covid. Moreover, it must be noted that diagnostic criteria are not static but dynamic categories that are constantly reformulated (See debates over the changing diagnostic criteria for cystic fibrosis (Hedgecoe, 2003; Kerr, 2005) resulting in the creation/resolution of diagnostic uncertainty for some patients at different time points; the changing diagnostic criteria for SARS-CoV-2 are a case in point.

In her recent discussion of diagnostic uncertainty and the act of diagnosis, Jutel (2021) observes that a failure to provide a diagnosis is seen as stemming from an individual clinician's indecision or cognitive biases rather than a failure of the classificatory system of medicine itself. This human-centred conceptualisation of diagnostic uncertainty is underpinned by a top-down view of diagnosis, which involves “… rapid reflexive hypothesis generation …” (Topol, 2019, p. 44), and relies on: a clinician's ability to perceive symptoms holistically, engage in intuitive thinking and use their tacit knowledge. In his recent analysis of the potential impact of AI on biomedicine, Topol (2019) observes that the hypothetico-deductive method of diagnosis invokes a number of cognitive heuristics (e.g. representativeness, availability and confirmation biases (Tversky & Kahneman, 1974), which means that clinicians attend to and seek out evidence (symptoms) that confirms their initial hypothesis. He notes that medical training prizes diagnostic certainty with the result that clinicians are taught to be overconfident in their clinical assessments and to avoid diagnostic uncertainty.

Constructing the failure to provide diagnoses as a human rather than a systemic failure may provide an epistemic defence for the practice of medicine (Jutel, 2021), but does little to help individual patients who remain undiagnosed and unable to access appropriate care. Three and a half million people within the United Kingdom have a rare disease (European Commission, n.d.), many of these are children who have a developmental delay. In many cases these children remain undiagnosed and their families are unable to access the right types of educational and social support. In this paper we look at the use of AI –phenotyping algorithms - as a potential tool to overcome diagnostic uncertainty in some of these cases.

## 2 Study design and methods

Using qualitative interviews, which focused on the case of CP technology, this empirical bioethics study specifically sought stakeholders’ views about the ethical issues raised by the use of computational phenotyping algorithms within the clinic. The next phase of the project will use these empirical data to inform a normative analysis that addresses questions such as how should we implement AI in clinical contexts? Here we report the descriptive data collected during the empirical phase.

### 2.1 Recruitment of participants

The *Minerva Initiative (MI)* is a research data resource (Minerva Image Resource) and an international consortium of commercial, clinical and academic researchers (Minerva Consortium) involved in CP research (Nellåker et al., 2019). The aim of the MI is to promote and facilitate CP research by enabling research collaboration amongst consortium members and to develop a secure platform for sharing digitised facial image data that may be used in the training of phenotyping algorithms.

Interview participants were recruited by email from the Minerva Consortium's membership list and by snowballing authors' and interviewees' contacts. Recruiting interviewees from the consortium and snowballing known contacts ensured that all interviewees were familiar with CP technology; overcoming the problem with much of the research (e.g. Lai et al., 2020) on the impact of AI, which frequently involve participants who lack familiarity with specific AI tools and thus, are often forced to consider hypothetical scenarios. Forty-seven individuals were sent an email invitation: two declined, one returned some comments by email, 20 (42%) were consented in writing and the remainder did not respond despite email follow-up. The final sample primarily comprised members of the Minerva Consortium, industry representatives and a rare disease patient support group spokesperson. Twelve of the interviewees had stated expertise in clinical genetics (with one involved in developing commercial clinical phenotyping applications); six in bioinformatics/data science/computational biology, and one each for industry, patient support groups, and neurosurgery. Geographically, participants were located in Europe (ten), the USA (six), Australia (three), and Africa (one).

### 2.2 Data collection

The data were collected during in-depth interviews, which took place remotely (telephone/Skype) in March and April 2019. Interviews were carried out by SB, lasted ≤60 ​min and were digitally (voice) recorded with interviewees’ consent. At the start of the interview the interviewees were asked an open-ended question about their views of CP technology and to reflect on its strengths and weakness. Open-ended questions based on themes identified in the AI and ethics literature followed, ensuring that all presented views on: the impact of the use of FR algorithms in rare disease research and healthcare; the use of photographic images versus other types of personal data in research, privacy and consent, issues around data access and sharing, particularly the difference between public and private initiatives in this area and the impact of data siloes, algorithmic bias and incidental findings (Hallowell et al., 2019).

### 2.3 Analysis

The interviews were transcribed and read through a number of times to enable the identification of recurrent themes within and between participants’ accounts. The method of constant comparison (Strauss & Corbin, 1990) was used to develop a coding scheme for analysis. Coding generated four overarching themes: *The impact of CP on the practice of dysmorphology*, *managing expectations about AI technology*, *trust in AI technology and the benefits and costs of using AI tools for diagnosis*. In this paper we focus upon what the interviewees regarded as the positive and negative impact - the benefits and costs - of deploying CP for diagnostic purposes.

## 3 Findings

### 3.1 The public benefit of using AI: democratising diagnosis

“[*CP] will have tremendous impact on diagnosing syndromes. And the real reason is, we are just terrible at it. As a group, we are just terrible and we cannot even diagnose the most common syndromes* ….” Clinical Geneticist P016

While most interviewees acknowledged that CP technology was still relatively immature (P010), there was widespread agreement amongst our interviewees that in the future the public benefit of using CP technology to diagnose rare diseases will be the provision of faster and potentially more accurate diagnoses for patients and their families. The evolution of diagnosis in dysmorphology over the last twenty years from a dependency upon expert judgement to genotype-assisted diagnoses has been swift, and interviewees reflected that the introduction of CP into the diagnostic pathway could take this to another level. They hypothesised that the use of CP as first line screening will: shorten the time to diagnosis, enable undiagnosed patients to gain a diagnosis, facilitate the prioritization of exome sequencing and may remove the need for specialist referral and molecular diagnostics in some cases.

*So my personal hope is that if you have the ability to take a photo of somebody and get some kind of answer in a clinic setting that you can get diagnoses much more quickly and you don’t necessarily have to go through this massive bottleneck, which is medical genetics.* Clinical geneticist P001

Because the use of this technology will potentially provide non-specialists with diagnostic capabilities, it was seen as democratising diagnosis by making it available to a wider patient group. As this patient support group spokesperson said:

*One of the things that I would like [about computational phenotyping] is that every patient has access to an equal level of service, and so I really like the idea that you go in, the computer does the same job for everybody regardless … the reality is that human interaction is not always the same for everyone. … I think it’s a huge problem [in rare diseases] because of course most clinicians will almost certainly never have seen somebody with your rare disease.* Patient support P003

The mainstreaming of dysmorphological expertise through non-specialists’ use of CP technology, was described as empowering non-specialist clinicians and promoting equality amongst health services users because it will provide patients, who are currently unable to access specialist care, with the chance of securing a diagnosis. This was flagged as particularly important in low- and middle-income and rural settings where a lack of human/medical resources and/or geographical constraints mean that many people have little or no access to specialist (dysmorphology) services or there are insufficient resources for genome sequencing. Clinical geneticists from sub-Saharan Africa, the USA and Australia speculated that in the future this technology could be used for remote triaging, saving patients from travelling to tertiary referral centres and the need to deploy specialists in remote regions.

*Being in Africa, we’re looking at a situation where not only are resources limited, distance is a problem, travel is a problem, access to healthcare is a problem. So there’s a lot of things that actually would be beneficial if we could have more distance involvement. … if one can really look at a process whereby one can screen or sift out patients via a more machine learning base, that requires again less hands on personal expertise.* Clinical geneticist P005

### 3.2 The personal costs of using AI to mainstream diagnostic skills

While the use of CP technology may expand the diagnostic skills of clinicians who lack phenotyping expertise, some interviewees also noted that AI technology could shift the locus of expertise in more radical ways, observing that non-clinicians could use it for self-diagnosis. Many of those working in clinical settings commented that parents of children with dysmorphic features often try to diagnose their child by going online and comparing their child to others who already have a diagnosis.

*But having said that, Dr Google also provides an enormous amount of information. And I’ve had a couple of children diagnosed by their parents by spending time on Google, …and because they are so much more invested in that particular case they can have the advantage sometimes of providing information that you don’t have. I guess the worry would be that if people then go down a self-diagnosis, self-management, you can end up in a little bit of a quagmire, again because of that issue of the importance of context.* Clinical Geneticist P005

They commented that parents’ tendency to (mis)diagnose their child often raises anxieties within the family, which are frequently difficult to allay.

*So when we see parents with children, some of them already went through the internet and came up with a sort of suggestion, and that’s not based on the face, but on the features in their child. …. Sometimes they are correct, but it’s more the other way around … And so when this sort of technique [CP] becomes generally available … yes, it will lead to confusion.* Clinical geneticist P002

Interviewees talked about a future in which parents could access and use diagnostic facial recognition algorithms, observing that while this may have benefits for some, it may also increase clinicians’ workload because they would need to manage false positive/uncertain results and the anxiety that may be generated.

*Yeah, I think the challenge with self-diagnosis is the false positives …. If you then say, well now [we’ll leave it with patients], I think it would just create an enormous amount of uncertainty and anxiety and false positives.* Clinical geneticist P004

At the same time as potentially up skilling non-experts – non-specialist clinicians and parents - interviewees talked about how this technology may result in the deskilling of experts - dysmorphologists. Some described dysmorphology as a dying art, observing that first-line molecular diagnostics is already deskilling dysmorphologists, who are at-risk of losing their clinical phenotyping skills. As this clinical geneticist said:

*I went to India and there are better dysmorphologists there than my colleagues here, simply because they have to rely on their dysmorphology knowledge more than we do. We can easily do genotype first and then start thinking about the result*. Clinical Geneticist P002

### 3.3 AI tools: assisting or replacing human diagnosis?

Interviewees went on to speculate about the future role of this technology in diagnostic decision-making, considering whether it will be used as a replacement or an assistive technology. In the first scenario, in which humans are removed from the loop, AI technology replaces expert clinical decision-making, in the second, where humans are retained; it reduces the margin for diagnostic uncertainty by providing external validation of clinical judgement. Both scenarios were considered by our interviewees, with some predicting that specialist dysmorphologists will no longer exist in the future because you will be able to take: …“*an ordinary facial photograph, feed it into a system and it will come out with an absolutely certain diagnosis”* (Paediatric geneticist, P007). Others were more circumspect, suggesting CP technology will be used as *“an expert tool”* to augment or assist expert diagnosis. As this clinical geneticist reflected:

*It’s very much an expert tool. So it’s not something that will replace an expert in that realm of medicine. But what it should be able to do is pull out patterns that we can’t see and therefore improve our diagnostic accuracy.* Clinical Geneticist P006

Most interviewees, like this clinical geneticist, were in favour of keeping human expertise within the loop, at least in the short to medium term, for as a computational biologist and former physician reflected, in addition to classifying or labelling patients, the act of diagnosis requires the consideration of which treatment/actions would be in the patient's/family's best interests.

*“I think many people would claim that this [CP] extends the capabilities of the physician. And the real art of being a physician, for me, I mean obviously you have to know how to manage knowledge and use your tools. I look at a computer as a new tool to help me manage knowledge that either was in my head and I couldn’t find it or just wasn’t in my head. But actually adapting that knowledge to the situation of your patient in front of you and figuring out the best course for them, …There are so many things that really, (laughs) at least now, are truly human that a machine cannot do.”* P013 Computational biologist

Others, pointed to the interactional element of diagnosis, commenting that the ways in which diagnoses are delivered and received are as important as receiving a diagnostic label and therefore, keeping humans in the loop who can interpret and explain an AI-generated diagnosis and what it means for treatment and prognosis is essential.

*… getting the diagnosis is only part of it. Actually, when you get given a diagnosis, the family needs to (a) understand the diagnosis and come to terms with the diagnosis … So I think the concern would be that, if your GP gave you – you’ve got this computer telling you this and your GP would not necessarily be the right person still to give you the diagnosis … but actually I think we would definitely feel there was a loss if the families then didn’t see somebody who had some understanding of the diagnosis ..I think still it’s really important that people are given the diagnosis in a sensitive and understanding way* Patient support *P003*

In summary, according to our interviewees, the introduction of CP technology into the clinic carries a number of costs; it may encourage self-diagnosis, which could lead to increased anxiety and, as a result, increase clinicians’ workloads. At the same time interviewees observed that the deployment of AI tools may result in the deskilling of dysmorphologists and dehumanise medical interactions. On the other hand, interviewees reflected that the use of CP tools can be seen as potentially beneficial because they may help clinicians who lack specialist expertise to reach a diagnosis and augment the skills of dysmorphology experts, thus, providing increased numbers of patients with access to a diagnosis. As the next section demonstrates, according to our interviewees, the potential of CP tools to increase diagnostic yield and reduce diagnostic uncertainty is based on the perception that they are better at classification tasks than humans.

### 3.4 Using AI to reduce diagnostic uncertainty: the benefits of technological objectivity

*So I think there are so many variables involved, especially with the more subtle phenotypes, that it’s all just a little bit of that and a little bit of this. And I think the deep learning algorithms can far better see the patterns and processing than what humans can.* Bioinformatician P015

All of our interviewees described algorithmically generated diagnoses as potentially more accurate, scientific, or unbiased, than a diagnosis based solely on clinical judgement.

*… currently it’s the case that you have this photo and then you go to a colleague and you say, “Do you think they look a little bit alike?” or whatever, and it’s very, very subjective, so not objective. And this [CP technology] is a sort of objective tool.* Clinical Geneticist P002

They stressed that CP is not subject to human biases, lack of experience, or emotion, and therefore, is not characterised by uncertainty or errors like human decision-making. As this paediatric geneticist said about CP applications: “*I have heard that it’s very helpful to people and it's more likely to be accurate than a whole room full of dysmorphologists, which is wonderful news.* (P007)*.* As far as our interviewees were concerned, CP is “objective”, “cleaner”, “scientific” and more efficient, and most saw the use of CP as potentially eradicating some of the ontological uncertainty or “guesswork” that lies at the heart of the diagnosis of rare disease. But while most interviewees were very positive about the diagnostic benefits promised by the use of this objective technology, they were also cautious and raised questions about the accuracy and objectivity of current CP technology.

### 3.5 The cost of employing AI diagnostics: the need for oversight of outputs and inputs

*I really like the computerised, automated, [diagnosis] because it feels very much more scientific than a person looking at someone else with their own prejudices. I like the cleanness of it. I think that my concern would be about how robust it was, how reproducible and how accurate it would be. I quite like the idea of it happening, because I like the idea that it’s done with no prejudice, it’s cleaner …* Clinical Geneticist P003

Many interviewees reflected on the reliability of algorithmic output commenting that perceptions of CP's reliability will need to be built up over time and will depend upon the accrual of external evidence regarding the accuracy of algorithmic-based diagnostic decisions. As a paediatric geneticist (P007) said *“Well, I think it's proof, accruing evidence, is what will lead to trust [in the algorithmic output].”* Indeed, interviewees speculated that the perceived reliability or trustworthiness of algorithmic output relies on the validation of AI-generated decisions using other types of knowledge like genomic data and/or expert knowledge in the case of rare diseases. Some argued that the input of human expertise was essential to accurate diagnostic decision-making arguing that in addition to facial phenotypes there were a range of other phenotypic features and subjective aspects of human–decision making that were necessary aspects of diagnosis in this context.

*There are aspects in human decisions that AI will never be able to substitute and they have to do with perceptions, feelings, they have to do with the personal interpretation of things, of both the operator and the person who sits in front of you. So it [CP] can be kind of, for me, you know, a side tool.* Clinical Geneticist *P010*

While recourse to other types of evidence may increase the perceived reliability and validity of a *C*P-generated diagnosis, many interviewees also pointed to another threat to developing reliable CP diagnostics, namely, the potential for algorithmic bias. As an Industry Spokesperson noted:

*You should definitely not trust anything without investigating. Because, especially with artificial intelligence, a very well-known phrase is ‘garbage in/garbage out’, right. The trust is established by looking, or basically believing or getting proof that the best technology is being used, but more important than that, that the data that’s being used to train the system is validated.* Industry spokesperson *P020*

Bias arising from the use of unrepresentative or biased datasets in algorithm training - the inputs - is a particular problem in rare disease research. Rare diseases are, as their name suggests, rare in the population and the lack of patient numbers always leads to questions about whether there are enough data available to train FR algorithms to identify a wide range of diseases in a wide range of people of different, genders, ages and ethnicities. As a data scientist reflected, not only are there biases in the types of data collected, but also how they are collected and processed:

*I think we necessarily have many biases in the data, in the sense that who is contributing is going to be biased by many things, right. It’s going to be which rare diseases are most amenable to facial imaging to begin with, maybe have less subtle types of phenotypes being captured. We probably have ethnic biases. We have biases on who is doing the photography: their particular technologies for doing the photography vary. We just have to be really careful not to bias ourselves, either in the collection or in the building of tools and resources or in the interpretation that a clinician might use and needs to be taking those things into account.* Data Scientist P018

Interviewees noted that questions about the representativeness of training datasets could lead to users questioning the reliability of algorithmic output, which may be exacerbated by the black box problem, or lack of transparency, about how CP algorithms work or which data were used to train them. Others acknowledged the increased accuracy of CP tools, but pointed out that if they were trained using unrepresentative data, then they may be unusable in certain populations.

*So that it’s highly possible that the objective measures are much more effective than we are. But again, they still have to be fed with enough information to cover different ethnicities, different ages, different – you know, so getting enough data for it to be accurate across the board. And build something else that only helps white people is not what we should be doing.* Clinical Geneticist P007

So while it was acknowledged that CP algorithms have the potential to benefit patients and their families by providing more objective diagnoses, it was clear that some interviewees were sceptical about whether this degree of objectivity had been, or could be, achieved, given the lack of data currently available for training. As this clinical geneticist reflected:

*… “often getting 20 individuals with a particular diagnosis is a challenge. And I think the other thing too is you also are inputting a variety of ages and individuals from a variety of different ancestral backgrounds that, I think, makes it challenging.”* Clinical Geneticist P009

Others regarded algorithmic bias as “a transient problem” (P012), which could be solved if and when more variable data, i.e. more ethnically diverse longitudinal data, were included in training.

In summary, our interviewees regarded CP tools as enabling users to overcome some of the subjective biases inherent in human diagnostic decision-making (e.g. psychological heuristics, emotions and a lack of skill and experience). At the same time they acknowledged that AI's objectivity is contingent and ultimately dependent on humans undertaking reliability and validity checks of algorithmic output and the training data.

## 4 Discussion

The stakeholder accounts generated in this study suggest that those who have an interest in the development of computational phenotyping tools are supportive of the deployment of AI for diagnostic purposes in dysmorphology. Like other commentators, they regard the use of AI tools (CP) as potentially widening access to health care by providing non-specialists with diagnostic capabilities (Lai et al., 2020; Drogt et al., 2022) and, as a result, more patients with a diagnosis (of rare disease). This has been seen as important in low - and middle-income countries (LMICs) (Kerasidou, 2021; Weissglass, 2022), particularly in places where genotyping methods are unavailable (Kong, 2019). Similar findings are reported in a recent literature review, which suggests that the majority of healthcare professionals whose work involves image interpretation (e.g. radiologists, pathologists and dermatologists) predict that the introduction of image recognition AI into the diagnostic pathway will improve patient access to screening, result in greater diagnostic confidence and save time (Scott et al., 2021).

### 4.1 AI tools: increasing diagnostic certainty

Overall support for the use of CP technologies in our study was grounded in the perception that AI-generated diagnoses have the potential to be more objective, efficient and accurate in contrast to human diagnoses, which are often characterised by bias or diagnostic uncertainty, particularly in the case of rare disease. As noted in the introduction to this paper, diagnostic uncertainty, or the failure to provide patients with a definitive diagnosis, is framed as a shortcoming of individual medical practitioners, who are seen as unable to make up their minds or as lacking requisite expertise (Jutel, 2021; London, 2019). This conceptualisation of diagnostic uncertainty is underpinned by a view of diagnosis as hypothesis testing, which draws on individuals’ tacit knowledge and experience (London, 2019) and is influenced by a number of subjective biases (Topol, 2019). In contrast, AI-generated diagnoses are bottom up and use inductive methods. AI tools provide diagnoses based upon objective or tangible evidence - digital data, which in the case of CP involves the analysis of facial images for feature similarities, comparative metrics and classification likelihoods. Our interviewees regarded the introduction of objective, value-free CP as a positive clinical development because they saw it as eliminating some of the uncertainty resulting from decision-making biases that are associated with human subjectivity (London, 2019; Topol, 2019). Like Scott et al. (2021), they saw the use of AI tools in the diagnostic pathway as promising increased objectivity, which manifests as greater diagnostic certainty and thus, increased diagnostic yield.

### 4.2 AI tools: the problem of reliability and validity

However, a minority of our interviewees noted that managing expectations about the use CP in the clinic is not so straightforward, for the idea that AI can and/or will produce definitive or infallible answers overlooks the fact that algorithms are socially constructed tools, which, although badged as more scientific and objective, are also subject to (human) biases. As has been argued elsewhere, the incorporation of fallacious assumptions about the representativeness of training datasets in CP may lead to misdiagnosis and consequently, disadvantage certain groups who may suffer from stigmatisation and discrimination and an inability to access care and support as a result (Hallowell et al., 2019; Kong, 2019). While we may try to avoid such outcomes by designing more transparent AI systems (AI HLEG, 2019; Morley, Floridi, et al., 2020), and ensure on-going dialogue between developers and users during the validation process (Winter & Carusi, 2022), the problem of the representativeness of training datasets is more intractable, particularly the curation of training datasets of rare disease phenotypes. As Kong (2019) notes, if we are to leverage the benefits of CP technology for diagnosis, then we need to access data from all human populations and curate training datasets of facial images that are as ethnically diverse as possible, otherwise there is the potential that CP will just exacerbate pre-existing global health inequalities.

However, the contextualised nature of healthcare (and healthcare data) means that algorithmic bias may be very difficult to expunge, at least in the short-term, so if we are to use these technologies, we need to consider how much algorithmic bias we are prepared to tolerate. This requires us to weigh the harms and benefits of using biased algorithms and raises questions such as, how do we balance the harms of a misdiagnosis or a missed diagnosis for some diseases or groups of patients against the benefits of providing a diagnosis for others, what criteria should we use: the number of people correctly diagnosed, the seriousness of the disease, the costs of treatments, lack of treatability and the increase in anxiety caused? Alternatively, to what extent does it matter if bias in the dataset means that diagnostic accuracy is higher in some patient groups, so that diagnostic yield is increased in, for example, some ethnic or some age groups, but lowered in others - could this technology result in further widening of preexisting social inequalities in healthcare or cause stigma and discrimination and what steps should we take to avoid this? As Kerasidou (2021) notes, addressing the bias problem in AI tools, such as CP, will require a much more concentrated and targeted effort to ‘fill the gaps’ particularly for populations in LMICs. Moreover, she points out, that this comes with its own practical ethical problems, namely, finding these populations, approaching them and convincing them to part with their data. Finally, Weissglass (2022) argues that even if it were possible to remove algorithmic bias and include data from populations from LMICs, then the lack of universal healthcare in many parts of the world means there may be few treatment options available even for those who receive an accurate and unbiased AI-generated diagnosis.

### 4.3 The art of diagnosis revisited

At this point it is important address the underlying assumption of this paper, namely, that one of the ultimate aims of healthcare is the provision of an accurate diagnosis. As noted above, diagnosis involves classification; determining whether X symptoms reflect underlying disease state Y, which in turn generates: a diagnostic label, and associated prognosis and treatment recommendations. Such narrow classification and prediction tasks are well suited to image recognition algorithms (Pierce et al., 2022) like computational phenotyping, which can be trained to correctly classify facial images as examples of particular phenotypes. Given the way these algorithms work, it is therefore unsurprising that our interviewees regard CP as providing as, if not more, accurate solutions to diagnostic problems in dysmorphology than (some) human counterparts. But, arguably this view of diagnosis is too simplistic, because as some of our interviewees noted, in many cases diagnosing a rare disease relies on balancing a constellation of competing (phenotypic and genotypic) features. Consequently, basing diagnosis on one type of features - facial phenotypes - may result in a misdiagnosis and bad treatment decisions, particularly if the technology is used by non-specialists who may lack knowledge about the disorder and the potential for complications arising from the presence of co-morbidities (Pierce et al., 2022). Moreover, many of our interviewees acknowledged that the act of diagnosis not only involves classification, but also is an interactional event. Indeed, the role of healthcare professionals is not only to provide patients with a diagnostic label, but also, and perhaps more importantly, to help them to understand, comprehend, and perhaps, accept the new reality that a diagnosis brings.

### 4.4 How should we use CP tools in the clinic?

This study suggests that those who are involved in developing/using CP technology regard it as having the potential to disrupt the diagnosis of rare diseases in positive and negative ways. Our interviewees, like those in Drogt et al.*'s* (2022) study, regarded CP tools as potentially providing non-experts with diagnostic skills which may make it easier for larger numbers of patients to acquire a diagnosis. However, at the same time as facilitating the mainstreaming of diagnostic expertise in non-specialists they recognised that the adoption of CP tools may result in the deskilling of specialists whose expertise has been established over a number of years. This generates a problem, at least in the short-term, for if CP tools come to replace human diagnostic decision-making for rare diseases we may be unable to provide external validation for these AI-generated decisions. Indeed as Winter and Carusi (2022) have observed on going input from expert users is essential during the validation of AI technology. This brings us back to a question that recurs again and again in discussions about the role of AI in healthcare. Namely, should we use AI as an assistive or replacement technology? We need to think carefully about this question prior to implementation in healthcare settings, particularly as history suggests that many technologies implemented in an assistive role often end up replacing human actors (Susskind & Susskind, 2015). Arguably, when deciding how and where we implement this technology not only do we need to question the assumption that the use of CP tools can *defacto* generate more accurate diagnoses given the questions raised about their validity and reliability, but also bear in mind that the act of diagnosis extends further than merely classifying and labelling a patients' symptoms (Jutel, 2021). Following our interviewees, and Drogt et al. (2022), we suggest that there is a argument for limiting the use of CP to an assistive role in which the AI-generated diagnoses continue to be ‘triaged’ and crosschecked with complementary biomarker and other data and by medical experts. This means, that the use of CP tools should be limited to healthcare settings containing clinicians who have existing expertise in dysmorphology and that non-specialists should receive extensive training and support in how to use these tools and be encouraged to seek external validation for their output rather than just deferring to the algorithm.

## 5 Conclusion

This study aimed to ascertain stakeholder views about the costs and benefits of clinical implementation of a particular AI diagnostic tool – computational phenotyping. Out interviewees indicated some ambivalence regarding the use of CP to diagnose rare diseases. While all regarded it as having the potential to democratise diagnosis by upskillling non experts, consequently making diagnosis more accessible to a wider patient group, they also expressed reservations about its clinical implementation. AI tools were perceived as making the diagnostic process less reliant on clinical judgement, thus undermining clinicians’ expertise and potentially compromising their ability to validate algorithmic output and identify algorithmic bias. Weighing these costs and benefits leads us to conclude that CP should be used to assist but not replace humans in the diagnostic process.

## Ethics approval

Ethical approval was granted by the 10.13039/501100000769University of Oxford, Oxford Tropical Research Ethics Committee (OxTREC - 549–17).

## Declaration of competing interest

The authors have no conflicts of interest to declare. This work was carried out while SB was a member of the Ethox Centre at the University of Oxford.
